# A World Beyond Transnational Corporations: Meeting Human Rather Than Corporate Need: Comment on "‘Part of the Solution’: Food Corporation Strategies for Regulatory Capture and Legitimacy"

**DOI:** 10.34172/ijhpm.2022.7540

**Published:** 2022-10-18

**Authors:** Fran Baum, Julia Anaf

**Affiliations:** Stretton Health Equity, Stretton Institute, University of Adelaide, Adelaide, SA, Australia.

**Keywords:** Commercial Determinants, Transnational Corporations, Markets and Health, Inequalities, Health Equity

## Abstract

This paper provides a commentary on Lacy-Nichols and Williams’ analysis of the emerging tactics of the ultraprocessed food transnational corporations (TNCs). Our paper provides an overview of the growth in power and influence of TNCs in the past three decades and considers how this change impacts on health and health equity. We examine how wealth inequities have increased dramatically and how many of the health harms are externalised to governments or individuals. We argue that human interests and corporate interests differ. The article concludes with a consideration of alternative ways of organising an economy that are more human centred and health promoting. We suggest five changes are required: improved measurement of economic outputs beyond gross domestic product (GDP); improved regulation of finance and TNCs; development of localised economic models including cooperatives; reversal of privatisations; making the reduction of economic inequalities a goal of financial policy. We consider the barriers to these changes happening.

## Introduction

Lacy-Nichols and Williams^1^ provide a very helpful analysis of the changes in the ways the food industry has sought to deflect criticism of its products and circumvent further regulation by seeking to become “part of the solution.” Their analysis is detailed and thoughtful and reveals much about corporate tactics. Here we seek to expand their analysis by examining the broader context in which transnational corporations (TNCs) operate and argue that the last three decades has seen a massive expansion in the power and influence of TNCs that poses a threat to health. We conclude with a consideration of what a post-corporate economic world might look like.

## Doubts About Neo-liberalism

The response of the food industry to public health calls for regulation in the forms Lacy-Nichols and Williams^1^ describe may also reflect their growing realisation that there is increasing questioning of the neo-liberal model of economic globalisation, including the dangers of overly powerful corporations. In 1995 Korten^2^ published “*When Corporations Rule the World*” where he warned of the danger of an economic system that encouraged the growth in power and influence of TNCs. In this book he outlined the ideal world of those urging further globalisation.

 In it, the world’s money, technology and markets are controlled and managed by global corporations, with a common consumer culture that unifies all people in a shared quest for material gratification. Perfect global competition among workers and localities offers investors the most advantageous terms.

 Corporations can freely act solely on the basis of profitability regardless of national or local consequences. As both individual and corporate relationships are defined entirely by the market there are no loyalties to place and community.

Wide ranging doubts about the power and influence of TNCs relative to nation states have increased since he wrote this book. A significant concern is that a class of ‘super rich’ has been created with disproportionate at fortunes compared with most of the world’s population. Oxfam has produced a series of reports on the increasing inequities in income distribution. The latest^3^ documents the rapid increase in wealth since the coronavirus disease 2019 (COVID-19) pandemic whereby the wealth of the world’s 10 richest men has doubled, while 90% are worse off. These ten men own more than the bottom 3.1 billion people and, since 1995, the top 1% have accrued almost 20 times the global wealth of the bottom 50%; with most accumulated through TNCs.

The other aspect of the changing global economy noted in the World Inequality Report^4^ is the extent to which private wealth has increased at the expense of public wealth, “due to deregulation, privatization, and increasing government debt.” The report further notes that TNCs and their shareholders “have been the main winners from globalization: their profits have boomed thanks to the ever-closer integration of world markets” (Chapter 8). Furthermore, “Between 1985 and 2018, the global average statutory corporate tax rate fell by more than half, from 49% to 24%” and there are no signs this trend is reversing. The report observes that tax evasion has increased from 1980s due to the rise of financial globalization and the liberalization of cross-border capital flows in the absence of any mechanism to tax or to effectively regulate.

In 2013 United Nations Conference on Trade and Development^5^ reported that global investment and trade are thoroughly entwined in international production networks and that 80% of trade now takes place in “global value chains” which are linked to TNCs. Milsom et al^6^ analyse the ways transnational tobacco, alcohol and ultra-processed food corporations use instrumental, structural and discursive power to ensure that no decisions are made which would inhibit marketing and sale of unhealthy products.

The rising power and influence of TNCs pose several threats to human health. Firstly, TNC have profit as their first consideration. The harms caused by highly processed foods TNCs are made clear by Lacy-Nichols and Williams.^1^ The same is true of the alcohol and tobacco TNCs. The fossil fuel industries which have continued to use carbon products despite the known dangers to the planet’s climate also threaten health. The *Lancet*^7^ notes that at the slow pace of carbon decarbonisation, it would take more than 150 years to fully decarbonise. Fossil fuel companies engage in political lobbying often through membership of representative organisations. This lobbying can influence government policy in ways that are unfavourable to health and equity.^8^ The loosely regulated media and communication industries can also cause harm. Social media has been associated with affecting young people’s mental health adversely.^9^ Facebook knew of the association between Instagram use and girls’ mental health and body image and suppressed the information.^10^ The cost of treating the harms caused by TNCs are often externalised to the public purse (see Table for example). Thus, the burden of chronic disease, environmental restoration, harm from alcohol, tobacco and gambling industries all place a burden on government services.

**Table:** Examples of TNC Types, Health Burdens and Externalities

**TNC**	**Health Burdens**	**Externalities **
Ultra-processed food and beverages	Increased obesity and chronic disease including cardiovascular, diabetes and dental caries.	Costs of treatment. Shorter life expectancy. Health inequities.
Alcohol	Increased cancers and cardiovascular disease, fetal alcohol syndrome, injuries and violence, including family violence.	
Tobacco	Increased cancers and cardiovascular disease.	
Fossil fuels	Main cause of global warming and climate change that threatens human survival. Destruction of ecosystems and decline of biodiversity as a result. Toxic wastes and industrial disasters including oil spills and tailings dam collapses.	Extreme weather events cause damage and human lives and livelihoods lost. Some countries’ existence threatened by human suffering and costs of flooding and sea level rise and massive bushfires. Threats to communities especially in low- or middle-income countries. Inequitable impacts within populations.
Social media	Mental health impacts.	Costs of treatment and/or individual and social harms resulting from a lack of appropriate treatment.
Practices common to most TNCs	Lobbying government and/or providing political donations to maintain products despite harms. Tax evasion. Profitability uppermost in decision making. Employing ‘health wash’ and ‘greenwash’ tactics. Creating ‘civil society organisations’ or ‘front groups’ to promote corporate views, and using public relations as a form of education. Using corporate social responsibility strategies to enhance the corporate image.	Threats to the democratic process. Reduction in health and social provision due to lack of revenue. Promoting values and outcomes inimical to population health. Lack of transparency over corporate operations leading to private interests prevailing over public interests. Influencing the level of health harming consumer purchases by generating greater credibility and trust. Entrenching self-regulation instead of government regulation with corporate compliance enforced to protect the public interest.

Abbreviation: TNC, transnational corporation.

In 1995, Korten made the basic point that “the human interest and the corporate interest differ” and since then neo-liberal globalisation has given TNCs more power and influence over governments and arguably exacerbated these differences. Korten^11^ later stated that the corporation’s internal economy is, paradoxically, a planned economy, with consolidation of power by large global corporations cementing central planning rather than the market economy espoused under neoliberal and globalist values; but to suit corporate interests.

## Alternative Economic Visions

The problems governments face in regulating the activities of powerful TNCs are shown by Lacy-Nichols’ and Williams’ work. The activities they document make effective regulation more difficult. TNCs can be nimble and agile in responding to attempts to regulate them, such as reassurances promoted by corporate social responsibility credentials. Governments typically are less agile and subject to extensive corporate lobbying. So perhaps the crucial question for public health raised over the past decade is less about how to regulate the TNCs (although this is an important question) but to determine what economic models might be closer to human interest. Some economists have called for degrowth economies.^12,13^ One of us (FB^14^) has proposed five action points for an economy based on human need drawing on the works of others^15-18^ who examined alternatives.

- *Measure what is treasured:* indicators of real economic progress are adopted, the assumption of economic growth is challenged, and the needs of people and the environment are counted, resulting in new indicators of economic and social progress. Several alternatives to gross domestic product (GDP) have been devised, including the human development index created by the United Nations to recognise people and their capabilities in assessing a country’s development. The genuine progress indicator includes the environmental and social factors that are not measured by GDP.
- *Better regulation of finance and transnational corporations: *regulate TNCs and financial markets to ensure that their activities create more societal benefits than costs and focus on health as well as wealth. Expediting the global agreement on changes to international taxation regulations for multinational corporations is one example.
- *Develop local economic activity:* local industries (including co-operatives) and alternative models of economic organization are developed and supported. Co-operatives are formed as non-profit enterprises and for the benefit of members rather than for corporate profits.
- *Promote public ownership of essential assets:* the mass privatization of essential services is reconsidered, and the benefits of public ownership asserted. Supporting the re-nationalisation of public infrastructure such as occurring in France with shipyards and planned for electricity is a positive step.
- *Reduce economic inequities as an explicit goal of fiscal policy:* fiscal policies such as progressive taxation including a currency transaction tax (‘Tobin’ tax) and ‘windfall’ profit taxes for fossil fuel TNCs are used as mechanisms to redistribute income and wealth and to fund health, education, welfare, and social security systems within countries. These should be implemented and viewed as an investment in health and well-being.

These points make it clear that the reforms required are political in nature, just as many past reforms in public health have required political will and action.

## Challenges to Implementation

 There is no doubt that reforms will be difficult due to the disproportionate power of TNCs. One example is the United Nations Treaty on Business and Human Rights which has been on the global agenda for over 40 years, with its 2011 ‘Protect, Respect and Remedy’ Framework for implementation. This remains a non-legally binding agreement facing ongoing business opposition, and with governments retaining close ties to business. Other obstacles include the success with which its proponents have enshrined neo-liberalism as an accepted for granted basis for national economies; the extent of the power and influence of TNCs over government agenda; and the relative weakness of civil society pressure for change. However, since the pandemic, which has steepened inequities, the voices of opposition to the current economic order are increasing and many see the transition to a zero-carbon economy as requiring one more focused on human need. Yet while governments put the economic rights of business ahead of human rights and public health we cannot expect a true commitment to radical change.

## Conclusion

 Reforming our economy away from the domination of TNCs seems like an impossible task given their powerful position in the current economic world order. Yet their focus on growth and profits requires systemic change as the planet’s sustainability depends on radical action. Public health needs to consider ways of regulating TNCs and also of replacing them with healthier alternatives. It is therefore imperative that public health advocates for equivalent representation at the policy table to that of business and thereby become a bigger part of the policy ‘solution.’

## Acknowledgement

 Professor Baum and Dr. Anaf’s time was funded by NHMRC Grant Number GNT2009323.

## Ethical issues

 Not applicable.

## Competing interests

 Authors declare that they have no competing interests.

## Authors’ contributions

 FB conceived the article and prepared the first draft. JA contributed to subsequent drafts. Both authors agreed on the final version.
